# Medical Sociology in Relation to Medical Colleges and the Profession

**Published:** 1906-11

**Authors:** R. R. Kime

**Affiliations:** Atlanta, Ga.; Gynecologist to Tabernacle Infirmary; Ex-President Tri-State Medical Society, Georgia, Alabama and Tennessee; Ex-President Georgia State Sociological Society; Ex-President Atlanta Society of Medicine and Atlanta Sociological Society


ATLANTA

### Journal-Record of Medicine

Successor to Atlanta Medical and Surgical Journal, Established 1855, and Southern Medical Record, Established 1870.

OWNED BY THE ATLANTA MEDICAL JOURNAL CO.

Published Monthly.

*Official Organ Fulton County Medical Society, State Examining Board, Presbyterian Hospital, Etc.*

VOL. VIII. NOVEMBER, 1906. No. 8.

BERNARD WOLFF, M.D., EDITOR, 319-320 Prudential Bldg.

M. B. HUTCHINS, M.D., BUSINESS MANAGER, 1007-1008 Century Bldg.

E. G. BALLENGER, M.D., ASSOCIATE EDITOR AND ASSISTANT MANAGER.

### ORIGINAL COMMUNICATIONS.

### MEDICAL SOCIOLOGY IN RELATION TO MEDICAL COLLEGES AND THE PROFESSION.*

BY R. R. KIME, M D., ATLANTA, GA.

Gynecologist to Tabernacle Infirmary ; Ex-President Tri-State Medical Society, Georgia, Alabama and Tennessee ; Ex-President Georgia State Sociological Society ; Ex-President Atlanta Society of Medicine and Atlanta Sociological Society.

This is a sociological age and we as a nation have reached that stage of evolution in which sociology is a very important factor in our development. When capital and labor are so at variance, strikes and trusts so common, insurance graft and impure food and meat so rife we are forced to believe our moral social fabric is diseased and needs purification. We are no longer a primitive rural people but fast becoming urban with its consequent accumulation of wealth and changed social conditions.

*Read at first meeting Fifth District Medical Society, Atlanta, Ga., Oct. 16, 1906.

As we increase in wealth, population and age as a nation we become diseased physically, morally and mentally, unless we utilize sociological principles for our improvement. To properly apply these principles to our social condition requires thought and study not only of one class or profession but of all classes and all professions. Each has its duties and obligations, but because of its intimate relation to the people, State and nation the medical profession has the greatest obligation. The sociological relation of the medical profession to our development as a people may be expressed in the title of this paper—Medical Sociology. The medical profession of this country in its evolution has passed through its primitive period and has now reached a sociological stoge which demands a study and investigation of our social conditions from a medical standpoint.

In the past the profession of this country has expended most of its energy in developing its own interests, each engaged in earning a living individually, and in meeting the demands of a rural population or comparatively new cities reinforced by new blood from rural sections.

The future has wider fields of study, investigation and usefulness for the medical profession. Crowding of population, increased wealth, more rapid intercommunication between ourselves and the world at large, develop new problems for study and investigation by the medical profession. As we advance along these lines, crime, vice, disease and degeneration will increase unless sociologic forces are put in motion to counteract same.

The physician of the past has confined himself principally to the study of man; the physician of the future must study mankind.

The greater part of a physician's work is with diseases of mankind rather than with man alone. Disease and degeneration in most instances are the result of association and conveyed directly or indirectly from one to another. The exanthemata, many skin diseases, the specific diseases, parasitic diseases, tuberculosis, typhus, typhoid, meningitis, even malaria and yellow fever, and much of surgery come in for sociological consideration. We are told preventive medicine will be the great work of the future and preventive medicine is based on sociology and sociologic conditions.

Much of disease and degeneration is due to immorality, then how shall we prevent such unless we study the cause? Immorality and disease can not be divorced.

Vice and disease are handmaidens. If you would know the law of cause and effect you must study both.

In the future the physician will occupy the most conspicuous place in the study and investigation of sociologic conditions. For this work and responsibility he must be educated not alone in the study of man, but of mankind in his various relations and associations. Here it is the medical college owes a duty and responsibility which should not be neglected or evaded.

Each and every medical college large or small should have a professorship or chair on medical sociology.

I maintain that the systematic study of medical sociology by the medical student in this day and generation is as important as any subject he is required to study.

The future physician can not be fully equipped for his duties and obligations without a study of this subject. In passing allow me to state that the small medical college properly equipped for efficient work has advantages the large colleges do not possess.

The small college with a chair on medical sociology filled by a physician of high moral character properly qualified for his duties would be a powerful factor in the social development of mankind and bettering the condition of the human race. So far as I know not a medical college in this country has even an instructor or lecturer on this subject.

We have a few medical books written on special medico-sociological subjects, such as "Social Diseases and Marriage," by Prince A. Morrow M.D.; "Diseases of Society," by G. Frank Lidston, M.D.; "Consumption and Civilization," by John B. Huber M.D.; and numerous others on the border-line of medicine and sociology. But none of these are used as text-books nor taught in our medical colleges. What medical sociology is taught comes in incidentally in teaching medicine and surgery from a medical standpoint rather than from a sociological.

We are told that the crusade against the "great white plague" depends principally on the physician, that the control of venereal diseases, social vices and instruction of youths on sexual matters belongs mostly to the medical profession, that the use and abuse of alcoholics depends on the attitude of our profession. Then we must consider patent medicines, pure foods, sanitary science and measures, local and general board of health work, the physical development of the child, medical inspection of schools, care of the defective class, the prevention of the spread of contagious and infectious diseases, the lessening of the death-rate from all causes and especially that of infant mortality and then we begin to have some idea of the scope of medical sociology. To these we should add the moral phase of the subject and the physical development of mankind, and last, but not least, the negro problem.

I have heard it stated in Atlanta, by highly educated intelligent smart men that there is no "negro problem," but I want to impress on you in burning words and write them upon your minds in colors as black as the Ethiopian himself that we have a *negro problem of great and serious import to this section of the country.*

Many of the phases of this subject belong to medical sociology. His physical, moral and mental characteristics should be studied. He should be considered in his social relation to the white race as a factor in the spread of tuberculosis and all contagious, infectious and venereal diseases.

He should be studied as a race in regard to alcoholism, drug addiction, insanity, crime and means evolved to prevent his most heinous of crimes, "rape."

It is said by some, "Let the negro question alone and it will settle itself." The echo answers, How? Humanity answers, How? As well might one say let the saloons alone—let tuberculosis alone—let ignorance, crime, vice and disease alone—all will settle themselves—but the echo! answers again, How?

I tell you that the race frictions of the past, the carnival of crime and rape and the "riot in Atlanta" are but low mutterings and rumblings of the great storms and destructions that are to come, unless sociological factors are awakened and control the future.

It is one of the supreme questions of the hour and no brave man can afford to shut his eyes, fold his hands and serenely wait the future crime, riot and destruction that is sure to come unless the races are separated or radical sociological measures are instituted.

We have briefly outlined some of the subjects that more strictly belong to the domain of medical sociology of which the enlightened physician of the future should know something and the medical student be instructed. If we add to these the study of the cause and prevention of crime, pauperism, insanity, then we ask who should be better prepared to investigate these subjects than the physicians? Criminology and penology offer vast opportunities for study and investigation by the physician.

In passing I would say that the profession should be awakened to their duty along these lines and especially as to the punitive system versus the probation system in handling criminals. The voice of the profession should be unanimous in favor of juvenile courts and the probation system for all criminals. Our present methods of handling criminals are schools of crime and a disgrace to an enlightened civilization.

In conclusion allow me to state that the student who enters medicine simply to make a living, or the physician who practices simply for the money, has a sordid, selfish view of the medical profession and is unworthy the name. Each and every profession, business or trade owes a duty to humanity.

The higher and nobler the profession the greater the duty. Each individual also owes a duty to humanity, the greater his knowledge and ability the greater his obligation. Then let us, like men and physicians, prepare to meet and fulfill that obligation to the best of our ability.

The literary colleges are teaching sociology, then let the medical colleges do their duty and teach medical sociology. In view of the fact that there is no systematic study of crime, etc., in the State, especially of the negro, except by members of their own race, I present the following for your consideration and adoption :

STATE COMMISSION.

In the interest of the State for the better development of the people with a view of securing a more harmonious relation of the races and lessening crime, vice and disease, be it

*Resolved*, 1st. That we indorse and urge the legislature of the State of Georgia to provide for the appointment and maintenance of a commission for the study and investigation of the primal causes of *crime*, *pauperism*, *insanity and mental degeneracy*.

2d. Said commission to be composed of two physicians, two lawyers, two ministers and two teachers selected because of their fitness and qualification for the work.

3d. It shall be the duty of this commission to study the above subjects from a scientific and practical standpoint as they pertain to the State and this section of the country in relation to both white and colored races and make annual reports of their work including suggestion as to the control and prevention of these evils.

4th. We urge the appropriation of at least $5,000 dollars by the legislature and so much annually as may be needed for the use of the commission that it may accomplish its work in an efficient manner, and disseminate such knowledge as will be of benefit to the various professions and general public of the State.

Adopted by the 5th District Medical Society.

---

Any one endorsing above resolutions, please cut out, sign with name and address, and mail to Dr. R. R. Kime, 509 English-American building, Atlanta, Ga.

